# Multi-tiered systems of support with focus on behavioral modification in elementary schools: A systematic review

**DOI:** 10.1016/j.heliyon.2023.e17506

**Published:** 2023-06-22

**Authors:** Jannik Nitz, Fabienne Brack, Sophia Hertel, Johanna Krull, Helen Stephan, Thomas Hennemann, Charlotte Hanisch

**Affiliations:** University of Cologne (Universität zu Köln), Germany

**Keywords:** Multi-tiered systems of support (MTSS), Elementary school, Systematic literature review, Tiered prevention, Behavior modification, Framework concepts, MTSS-B

## Abstract

Multi-tiered systems of support (MTSS) are effective in addressing challenges in schools through a tiered system of support and diagnostics. A broad field of research has developed over the past 50 years. This systematic literature review aims to provide an overview of MTSS quality, outcomes, and characteristics in elementary education research. The review includes international studies and focuses on MTSS approaches that integrate behavior modification. After searching several databases, 40 studies published between 2004 and 2020 met the criteria for closer examination. The review presents study characteristics and theoretical references of different MTSS, including location, time, sample, study design, outcome measures, groups involved, interventions, and effects. In summary, MTSS have been found to be effective in elementary schools internationally, particularly for behavior change. Future studies should investigate the interactions between interventions within the school setting and involve teachers, school staff, and stakeholders in MTSS development to improve the system's coherence and effectiveness. It's important to note that MTSS have a political dimension that affects implementation and sustainability and can impact society by improving school experiences and reducing negative behaviors.

Multi-tiered systems of support (MTSS) are comprehensive frameworks that provide individualized support to students through a tiered system of evidence-based interventions [1,2]. Their aim is to improve positive school experiences and decrease negative educational outcomes [3].

The last 50 years have seen a significant amount of research on tiered prevention systems, with a focus on its implementation and effectiveness [4,5]. One of the main reasons for this is that schools continue to face challenges in addressing issues related to disruptive behavior or supporting students with emotional and behavioral disorders [[6], [7], [8], [9]]. Frameworks such as Response to Intervention (RTI), School-Wide Positive Behavioral Support (SWPBS), and Positive Behavioral Interventions and Supports (PBIS) have been shown to be effective in addressing these issues in inclusive elementary schools [e.g., [10,11]].

These frameworks have been shown to be effective in addressing behavior issues and promoting academic success in inclusive schools. Various systematic reviews [5,[10], [11], [12], [13], [14], [15]] have addressed some of the concerns raised by schools and researchers regarding the development of MTSS, but an overview for elementary schools internationally is missing. Specifically, elementary schools have certain requirements for the design of MTSS [16,17]. The effectiveness of the approaches and interventions, as well as the design of the different systems, can serve as a starting point for the development of further systems.

## 1 Multi-tiered systems of support

Multi-tiered systems of support (MTSS) create a common context of action among diverse school stakeholders to support the academic and social-emotional learning of all students. This is accompanied by a positive influence on other groups of people at school such as teachers, staff, and social workers. MTSS are preventive approaches and intended to reach all students in a school system [18]. The rationale is to dovetail research and practice so that empirically validated interventions are implemented into the MTSS to ensure the best possible support for students [1]. This process is supported by student diagnostic data, so MTSS can be seen as an approach that conditions school-wide impact and relates to whole school culture [2].

MTSS are widely spread around the world and can be found in different educational systems such as preschool, elementary school, secondary school, or high school [e.g. [12,[19], [20], [21]]]. The underlying mechanisms are mostly the same and they differ in terms of content and design. In general, MTSS create alignment and pedagogical coherence in a system in terms of support and professionalization. Reasons for implementing a MTSS are mostly legal entrenchment of tiered support systems, empirical evidence, and teacher and school overload. The impact of various forms of MTSS has been empirically proven in studies, so that the mechanism and effectiveness of MTSS will first be presented without focusing on elementary school. This is important to present a holistic picture of tiered support, which will finally be specified with a focus on elementary school. This focus is necessary because of the existing literature, which does not always include all the different subtypes of MTSS in the comparison. However, the special needs of elementary schools with respect to MTSS and the increasing rise of behavioral problems in school make this area of research necessary from an educational-practical perspective [7,[22], [23], [24]].

### 1.1 The tiered system

One essential component of MTSS is the three tiered system: MTSS usually include the following three levels/tiers: (1) *primary*, (2) *secondary*, and (3) *tertiary* [25]. In each of these tiers, a different form of support is provided. This intensity increases from Tier 1 to Tier 3. MTSS often focus on academic and learning-related factors [26], but also address emotional and social behavior [27]. In this context, it is important to note that the academic and behavioral needs of a student may be addressed at different tiers within a MTSS. As in the beginning said, central to MTSS and its tiered system is the collection and analysis of school-related diagnostic data. The data may vary, and it includes behavioral, academic learning, or social processes, which leads to the determination of interventions. Thus, interventions on different tiers are planned based on the diagnostic findings.

Tier 1 interventions should ideally reach all students and include evidence-based interventions. Behavioral interventions should be oriented toward positive reinforcement and consequences so that the school experience is positive and social-emotional learning is enabled. Academic interventions focus more on instructional structuring measures such as classroom management or differentiating measures. For students who do not respond as expected to Tier 1 interventions, Tier 2 interventions are provided. Along the approximate prevalence numbers, this is about 10–15% of the class [24,28]. The goal is to offer individualized interventions for these students based on their needs. The interventions applied vary depending on the MTSS and the students' conspicuousness but are guided by objective criteria and a diagnostic process. If the more specific interventions at the second level are not successful and cannot positively influence the target behavior, help is provided on a third tier. This third tier includes 1%–5% of students and is characterized by interventions that focus more on the student's problem behavior or academic situation [28]. Interventions on the third tier often take a systemic perspective.

### 1.2 Different types of multi-tiered systems of support

Seminal writings that influenced MTSS in terms of academics, and others that influenced MTSS in terms of promoting social behavior emerged over time [29]. The most important and well-known concepts are: School-Wide Positive Behavior Support (SWPBS) [30], Response-to-Intervention (RTI) [31,32], and Positive Behavior Interventions and Supports (PBIS) [25,33]. From these, different subtypes of MTSS have been developed, focusing on different problematic areas in academic learning or behavior. For example, some MTSS have been recently developed, focusing on traumatized students [13] and school safety [34]. Further specified, there are MTSS for different levels of education (e.g., elementary school, middle school, high school).

### 1.3 Effects of multi-tiered systems of support

The body of research on the various positive effects of different MTSS forms in different school systems is quite extensive. Various studies have demonstrated the effectiveness of different MTSS forms and report positive effects on variables over the different systems and school levels. The most prominent are school or classroom climate [e.g. [10,35]], disruptive behavior [e.g. [36,37]], and internalizing behavior problems [e.g. [27,38]]. Emotional and behavioral disorders such as attention-deficit/hyperactivity disorder (ADHD) are also the subject of research within MTSS. Here, positive effects on the expression of the symptoms of ADHD are reported [39]. Additionally, the connection and relationship between academic performance and student behavior is also research topic in school research focused on MTSS. Findings indicate that the MTSS that combine behavior change and reading interventions have larger effect sizes on reading skills than models without any behavioral interventions [40,41]. Studies also described high effectiveness of MTSS when implemented as a schoolwide systemic approach. In this, it can be an efficient and effective method for improving students’ academic and behavioral learning [11,42].

Some studies have explicitly addressed teacher health, focusing on the quality of teaching and the safety of school systems [e.g. [[43], [44], [45], [46]]]. These factors can be targeted and effectively influenced through MTSS [47]. It is important to say here that often the focus is not explicitly on teacher behavior. The improvement of teacher health is achieved through less problematic student behavior and increased classroom structure.

Another relevant component of research related to MTSS at the teacher level is burnout, which can have various negative effects on classroom activities, as well as the relationships with students. Teachers who are overworked or suffer from burnout are less tolerant of student behavior problems and have more conflictual relationships with their students [48,49]. This can further increase stressful experiences [50]. Another issue in this context is teachers' self-efficacy, which is based on the basic theoretical concept of social-cognitive theory [51]. This focuses on the development and exercise of human agency. The core proposition is that people can exert some influence over their actions [[51], [52], [53]].

Positive teacher self-efficacy is also associated with positive student outcomes. For example, more self-efficacious teachers use more creative and elaborate strategies, thus creating a positively challenging learning environment [54]. In this context, various studies dealing with different school levels conclude that the implementation of MTSS has a positive effect on the teachers' well-being and on their experience of self-efficacy [[55], [56], [57]]. Furthermore, tiered support in schools can reduce the burnout rate and symptoms [58].

### 1.4 MTSS in the elementary school

Looking at the previously reported effects of MTSS across grade levels and across schools, it is worth taking a closer look with respect to the needs of students in elementary school. Often, the initial focus of MTSS in elementary schools is on basic academic skills in reading, writing, and math [59]. Accordingly, diagnostics around the development of these skills are also important. Continuous monitoring by teachers coupled with feedback to students is a core component of tiered support [60]. This diagnostic monitoring is proven to be effective and is ensured by tiered support. A challenge for teachers at the primary level is the area of learning to learn. Academic strategies must be developed and consolidated, which can be supported by the MTSS implementation [61]. Furthermore, social-emotional learning is another important component at the elementary level. This means a major developmental concern of students in the school entry phase is the shaping and development of social and emotional strategies, the development of knowledge about emotions, and how to deal with them [62]. For this reason, MTSS such as SWPBS focus not only on academic processes, but also address behavior modification. As the rate of students with behavioral problems and emotional and behavioral disorders in the classroom increases in terms of any inclusive processes, the relevance of these systems and its pedagogical interventions increases [7,[22], [23], [24]]. For this reason, this review focused on MTSS that do not purely address academic learning but have integrated behavioral dimensions as well, which is described in more detail below.

### 1.5 Objectives of the presented systematic review

As described, there is a wide range of different studies on the topic of MTSS. This review aimed is to contribute to the field around the needs and research in the context of elementary schools. Different types of MTSS have already been summarized in previous systematic reviews and investigated with different focuses. Three papers summarize studies in the context of SWPBS and elaborate effects and implications for student behavior and staff outcomes [5,14,15]. The review and metanalyses by Lee and Gage [11] summarizes existing reviews and metanalyses with supplementation from additional studies and provides an up-to-date overview of existing research on SWPBS. Other reviews focus on individual constructs or the different tiers and their interventions [e.g. [10,12,13]]. Another two review focuses on preschool and different study designs [21,63].

In summary this means existing review papers focus on different areas of the topic and do not provide a comprehensive current state on elementary schools alone. However, the needs of MTSS systems are special due to the school environment in elementary schools: the classes are often very heterogeneous and the teachers, in addition to teaching literacy and basic skills, have a major pedagogical role to provide structure and security [e.g. [64]]. Furthermore, students enter the first institutionalized educational setting of their lives, which can set the course for many other educational experiences.

In addition, there is a lack of international overviews that include MTSS forms whose design and implementation are comparable to SWPBS, PB(I)S, or RTI but are arranged differently. This comes because different school systems in international comparison ensure that the characteristics and focus of MTSS may differ. However, this view is relevant for several reasons: Many emotional and behavioral disorders are developed in early childhood, as well as in the early school years, and they manifest themselves in the following years if they are not addressed in a supportive manner, for example, in the school context [65,66]. In addition, the number of students with emotional and behavioral disorders in the general school has been on the rise [67]. Moreover, since all children must be educated in the elementary school, this is a place where teachers should be sensitive in a particular way to special needs of any kind (emotional and social, motor, cognitive, learning, and so on). Another aspect is the inclusion of the affected groups of people in the context of the support. As a systemic approach, MTSS should include all persons involved and integrate them into the support. The factor of multimodality is therefore of great importance. Thus, various aspects justify the relevance of MTSS, particularly in elementary school. Accordingly, the following questions can be derived:

Research Question 1 (RQ 1)

What are the characteristics of MTSS in elementary education internationally?

a. What are the different types of MTSS and their origin that have been evaluated in different countries internationally?
b. What kind of pedagogical interventions in the context of behavior are used in the different tiers of MTSS?
c. Which groups of persons are involved in MTSS?

Research Question 2 (RQ 2)

What are the characteristics of empirical quantitative research on MTSS in elementary education internationally?

a. What is the current state of international empirical quantitative research on MTSS?
b. What were the main variables (outcome measures) collected to assess the effectiveness or implementation of the MTSS?
c. What are the effect sizes reported for different MTSS?

## 2 Method

### 2.1 Search procedures

An unsystematic search using the snowball system was conducted at the very beginning of the development of the search algorithm. Here, various search terms from the later search matrix were employed to identify survey papers and basic articles. At the end, the following search matrix was employed to conduct systematic literature search via EBSCO Host in the American Psychological Association (APA) PsycArticles, APA PsycInfo, ERIC, and MEDLINE databases:

SU tier* OR AB (“MTSS” OR “RTI” OR “PBIS” “PBS” OR “SWPBS” “SWPBIS” OR “response to intervention*” OR “positive behavio*” OR school-wide OR “integrated approach”)

AND AB (*school* OR *grader* OR “primary” OR pupil* OR child* OR learner OR kid* OR student* OR educat* OR elementary) NOT AB (college OR “high school”). The search was repeated by different people.

In addition, the FIS Bildung was searched using a translated German search matrix. The search was conducted in October 2021 and was rerun shortly before the review was completed. No articles were added during this process. In addition, various existing reviews and meta-analyses were searched for suitable articles and studies.

### 2.2 Study selection

Rayyan [68] was used for screening the articles. After this, the data were transferred to Covidence [69], which was used for full-text screening. Covidence was also used for data extraction. The process of abstract screening and full text screening was done by four different project members with an agreement of 91.3% and an interrater reliability of k = 0.64, which is considered substantial agreement [70]. Conflict cases were discussed in several joint meetings with all project members. In this manner, we were able to ensure consistent coding. Peer-reviewed German and English studies were included that had a quantitative empirical research design, concerning MTSS, and reported a sample of students in an elementary school. Elementary schools were defined as those school systems that explicitly provide initial school education up to a maximum of 7th grade. Studies that explicitly reported a sample of preschool were excluded. In this context, the type of school was irrelevant. No distinction was made between public, private, urban, suburban, and rural schools. Along the selected studies, 100% agreement between coders was achieved. Thus, studies that had an unclear sample or had a sample across school types were excluded. In addition, behavioral interventions had to be integrated into the MTSS. Systems that focused purely on academic learning were excluded. After the successful full text analysis and the exclusion of further texts, 25% of the studies in the extraction process were coded by three project staff members and the remaining 75% by two project staff members.

**Fig. 1:**
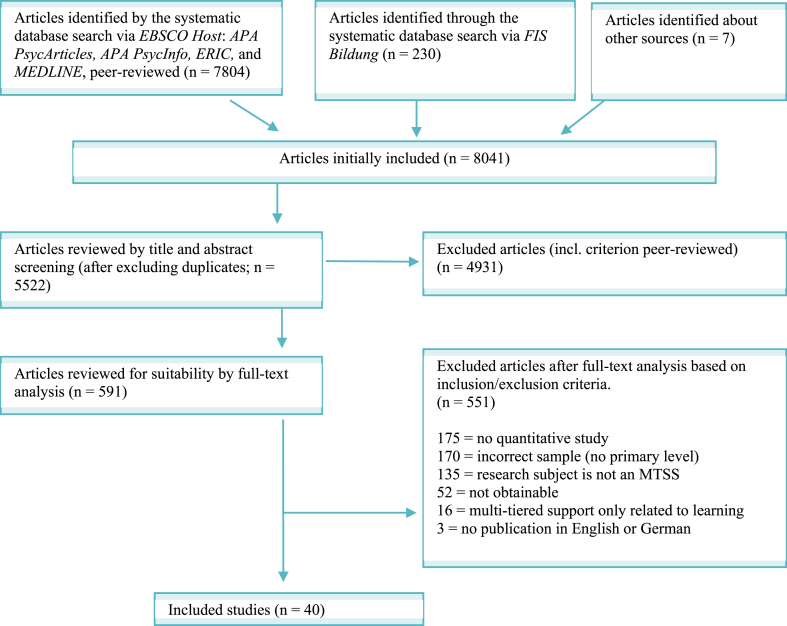
PRISMA Flow Diagram of included and excluded studies.

In total, the search yielded 8041 hits. During the abstract screening 4931 articles were excluded. Of all the articles reviewed 591 articles were included in the full text screening. Finally, N = 40 studies were included in the review. A detailed overview of the included articles and the search can be found in the PRISMA diagram (Fig. 1), which was created according to the latest standards [71].

### 2.3 Coding process

The included studies were subsequently subjected to a comprehensive coding process. Almost all research questions except RQ2a required the coding of different variables. Therefore, the following variables were coded: *Reference, MTSS Form, Involved Persons, Location, Design, Interventions, Outcome Measures, Main Results*. Due to complexity the definition of the coded variable *Outcome Measures* can be found in Table 6

For reporting the effects, the following variables were also coded: *Cases, Change (Mean), Significance, and Effect*. These variables were coded by at least two project members per study. At the end this was controlled for by at least one other person. The same process was done for the different interventions of the different tiers of the MTSS. Here the variable *Interventions* was coded, which can be traced in Table 2 including definition and listing of the individual interventions. Regarding the quality of the coding, it can be said that the close cooperation of the coders and the definition underlying the variables provided a high level of agreement from the beginning. The control of the extracted information by a third additional person further ensured the consistent coding. An exact listing of each variable and its underlying definition can be found in the Supplemental Material (Supplement - Coding for Variables).

**Table 1:** MTSS in elementary school.

Reference	MTSS	Involved Persons	Location	Design	Interventions	Outcome Measures	Main Results
Albrecht, Marthur, Jonas, & Alazemi (2015) [84]	PBIS	S, T, SSW	USA	QED (3 y)	T1 = School-wide social skill training (SST) T2 = Targeted SST & anger control training T3 = Conflict intervention & problem-solving training	- Attendance - Time away referrals - Discipline referrals - Academic scores	- Increases in attendance - Reductions in time away and disciplinary referrals, mixed results in academic achievement scores - Data indicated changes in the climate of the school
Algozzine& Algozzine (2007) [78]	SWPBS	S, T	USA	RCT (1sy)	T1 = Establishing unified attitudes T2 = Fostering unified expectations T3 = Implementing unified correction procedures T4 = Sustaining unified team roles	- On task behavior - Off task behavior	- Helped teachers to shift their focus from managing problem behaviors to teaching academic and behavioral content - Helped students to improve their attention to important classroom tasks
Betters-Bubon, Brunner, & Kansteiner (2016) [85]	PBIS	S, T, P, ST	USA	QED (6 y)	T1 = Sheltered instruction observation Protocol (SIOP) T2 = e.g., CICO T3 = Interventions for youth and families in crisis	- ODR	- Change of level of ODRs between different groups of students - Cultural equity through PBIS
Blumenthal & Voβ (2016) [86]	RIM	S, T, P	Germany	QED (4 y)	T1 = Standards for quality teaching T 2 = Individual support plan T 3 = Support in small groups and differentiated individual support	- Mathematics - Cognition - Phonological working memory - Emotional-social development - (Written) Language	- Better performance in emotional-social development, prosocial behavior, performance in math & German & social integration - Successful support for students with special needs
Borgen, Kirkebøen, Ogden, Raaum, & Sørlie (2020) [79]	N-PALS	S, T, ST	Norway	QED (5 y)	T1 = Defining and teaching consistent behavioral expectations & recognizing students for expected and appropriate behaviors T2 = Time-limited small group instruction T3 = Individual interventions and support plans	- Classroom noise - Bullying - Academic performance - Well-being - Pull-out-instruction - Special education - Student composition controls - Parents level of education & earnings - Immigrant background - Interactions between school county and school cohort	- Reduced classroom noise - Important differences in outcomes between the intervention and control schools, independent of the implementation of SWPBS - A credible design like is essential to handle such school differences.
Bradshaw & Pas (2011) [87]	PBIS	S, T, PS	USA	QED (2 y)	n.a.	- Training in PBIS - Adoption of PBIS - Implementation - Suspensions - Student mobility - Student to teacher ratio	- Correlation of different factors concerning the school level, the receipt of training, and the program adoption - Important role of school psychologist - Implementation needs more time than often considered
Bradshaw, Pas, Goldweber, Rosenberg, & Leaf (2012) [88]	PBIS	S, T	USA	RCT (3 y)	n.a.	- Teacher efficacy - Student outcomes - Including rate of special education service - Academic performance	- Positive effects on teacher efficacy and student outcomes, including rates of special education service use and teacher-reported academic performance
Cheney, Blum, & Walker (2004) [83]	SPBS	S, T, P, ST	USA	QED (3 y)	T1 = Schoolwide Support for Students with or at Risk of EBD T2 = Classroom and Individualized Support T3 = Family and Interagency Collaboration	- Social skills - Problem behavior - ODR	- Student's social skills improved - Problem behaviors decreased - Social skills and behavior problems were found to be related to the number of ODR's students received
Debnam, Pas, & Bradshaw (2012) [89]	SWPBIS	S, T	USA	QED	T2 = CICO, behavior charts/contracts, social skills groups, academic interventions T3 = FBA	- Monitoring and decision making - Responding to behavioral violations	- High implementation quality - Most of the schools use FBA to select intensive interventions.
Debnam, Pas, & Bradshaw (2013) [90]	SWPBIS	S, T, ST	USA	QED	n.a.	- Age & Sex - Implementation Quality - Some scores of the Organizational Health Inventory - Foundations - Targeted interventions - Individualized interventions	- Schools' organizational health played an important role in staff members perceptions of administrator support for SWPBIS and Tier 2 and 3 interventions & the implementation quality of these interventions did not. - Administrative perceived support for Level 2 and 3 interventions depending on the role of staff in the school
Ervin, Schaughency, Goodman, McGlinchey, & Matthews (2006) [91]	EBI	S, T, PS	USA	QED (4 y)	n.a.	- ODR - Days spent in disciplinary consequences - Implementation quality - School wide information systems - Basic Early Literacy Skills	- Addresses student behavior and reading from a public health prevention model - Providing a systematic continuum of supports and interventions - Establish an interactive and self-checking process to guide systems change and improvement
Ervin, Schaughency, Matthews, Goodman, & McGlinchey (2007) [92]	SWPBS	S, T	USA	QED (4 y)	Tier 1 = social-skill expectations (school rules etc.), Cool Tools	- Implementation quality - ODR - School-wide information systems	- Reductions were noted in the number of student discipline problems
Feuerborn & Tyre (2012) [93]	SWPBS	S, T	USA	QED (1 y)	n.a.	- Benchmarks of quality (BoQ) - Behavioral violations - Detentions and suspensions	- Positive changes in school-wide behavior and discipline practices
Franzen & Kamps (2008) [72]	SWPBS	S, T	USA	CS (2sy)	T1 = Teacher Training T2 = Give Me Five & Token systems T3 = intervention plans for school breaks	- General disruptive behavior - Inappropriate use of equipment, physical contact & verbal behaviors - Physical aggression - Active teacher supervision - Teacher reprimands	- Decreases in disruptive behaviors across three grade levels and increases in active teacher supervision
Goodman-Scott (2013) [94]	PBIS	S, T, ST, P	USA	MM (3 y)	T1 = Token economy system, learning school rules, classroom lessons T2 = Small-group intervention, CICO T3 = Specialized and individualized intervention, CICO	- Discipline records - ODR - Staff and student feedback	- School counselors play a critical role in the implementation of PBIS in their schools. - PBIS can be marketed as a cost-effective, efficient intervention
Greulich et al. (2014) [95]	RTI	S, T	USA	MM	Tier 1 = Reading Tier 2 = Group sessions (Imagine it!) Tier 3 = Smaller group sessions, Early Interventions in Reading	- Phonological awareness - Expressive vocabulary - Untimed reading - Fluency composite - IQ Teacher rating of reading - Social and problem behavior rating	- Teacher ratings of behavior and academics, accounted for a small amount of additional variance (3%) in group membership - Inadequate responders demonstrated physical and verbal task avoidance and displayed emotions of hopelessness and shame
Hill & Flores (2014) [96]	PBIS	S, PST	USA	CS (21 d)	T1 = Gotcha tickets T2 = CICO T3 = BIP & FBA	- Peer recognition for meeting expectations - Perception of satisfaction with the program - Amount of positive comments for successful task completion during instructional interactions with students	- Positive climate, a sense of empowerment, an understanding of expected behavior, and professional development through modeling and active mentoring - Enhanced teacher retention - Reduced stress and isolation - Increase of frequency of the target behavior: writing positive comments about others
Kelm, McIntosh, & Cooley (2014) [97]	PBIS	S, P, T	Canada	QED (2 y)	T1 = Explicit instruction, positive reinforcement T2 = Small-group social skills instruction, mentoring T3 = n.a.	- Problem behavior - Academic achievement - Reading - Math - Writing - School safety - Descriptive feedback	- Positive academic and behavioral outcomes for students - Increased perceptions of safety - Understanding of school expectations - Decreased perceptions of bullying - Reducing of ODRs - Increase of academic achievement - Establishing a positive school climate
Lane-Garon, Yergat, & Kralowec (2012) [98]	PBIS	S, T, P ST	USA	QED	T1 = Strive for Five/Mentored peer mediation T2 = Mentored peer mediation	- Perspective taking - Empathy - Conflict strategy	- Significant differences found on perspective-taking and empathy scores of the student mediators versus nonmediators
Lane, Kalberg, Bruhn, Mahoney, & Driscoll (2008) [99]	SWPBS	S, T	USA	QED (107 d)	n.a.	- Behavior disorder - Risk status - Access to reinforcement	- Illustration of various methods for assessing treatment integrity - Exploration of the possibility of using systematic screening tools - Identification how different types of students respond to SWPBS
MacLeod, Hawken, O'Neill, & Bundock (2016) [73]	FBA	S, T	USA	CS (3w)	Tier 2 = CICO Tier 3 = Function based intervention, individualized intervention	- Problem behavior - ODR - Social validity	- The combination of secondary and individualized function-based interventions effectively decreased problem behavior for all participants - Teachers and students rated the interventions as acceptable and effective.
Martella et al. (2010) [100]	PBS	S, T	USA	QED (17w)	Tier 1 = Four–fiveive broad expectations Tier 2 = Group social skills training, formal and informal friendship groups, classroom placement, CICO Tier 3 = Individualized wraparound services, functional behavior assessments (FBAs), special education support, one-on-one counseling + social skills training, CICO, behavior intervention plans (BIPs)	- ODR - In-class reporting - reports to parents, staff, etc.	- ODR were not representative of teacher recordings of classroom behavior. - Weak relationship between teacher recordings of disruptive classroom behaviors and ODRs
Nelsonet al. (2018) [74]	CW-FIT	S, T	USA	CS (1sy)	T1 = Social skills lessons, points, praise & goals T2 = Self-management strategies	- Task-related behavior - Proportion of praise and blame	- Results indicated student on-task behavior significantly improved. - Teachers were able to implement CW-FIT with fidelity. - Teachers increase their praise-to-reprimand ratios. - Teachers and students found the intervention to be socially valid.
Pearce (2009) [75]	RTI	S, T	USA	CS (2 y)	T1 = cCassroom & building level approaches T2 = Applied behavioral analysis, cognitive behavioral interventions, social skills training, counseling, parent involvement T3 = Special education program	- ODR - Maladaptive behavior	- Positive effects for improving student behavior, being accepted by education staff and families and the children themselves
Sanetti & Collier-Meek (2015) [76]	MTIS	S, T, P	USA	CS (27 Sessions)	Tier 1 = Feasible strategies, consultation, Direct Training Intervention Manual Tier 2 = Implementation support strategy, more intensive treatment integrity (1–2 sessions), role play, implementation planning Tier 3 = Intensive support on ongoing basis, performance feedback, participant modeling in 2 sessions	- Treatment integrity - Student outcomes - Implementation support duration	- All teachers responded to supports. - Response magnitude was different - Higher levels of treatment integrity were associated with fewer disruptive behaviors. - Duration of support strategies in-creased across tiers. - Increasing levels of implementation supports and subsequently higher levels of treatment integrity were associated with decreases in the number of disruptive behaviors.
Savage, Lewis, & Colless (2011) [101]	SWPBS	S, T, P, ST	New Zealand	MM (6 y)	n.a	- ODR	- Positive impact of SWPBS on behavior - Disciplinary reduction - Successful implementation thru schools' readiness, student empowerment, community input, professional learning, and evidence-based decision making
Sherrod, Getch, & Ziomej-Daigle (2009) [102]	PBS	S, T	USA	QED (2 y)	Tier 1 = Lessons by homeroom teachers, targeted behavior referrals, academic behavior monitoring Tier 2 & 3 (targeted students) = support group	- Behavior referrals - Discipline referrals - Student behavior	- Behavioral referrals decreased - Positive behavior ratings reported increased - 60% of those students reduced their discipline referrals to zero
Sørlie et al. (2018) [103]	N-PALS	S, T, P	Norway	QED (3 y)	T1 = Prosocial skill training, systematic praise, classroom management strategies T2 = CICO T3 = Individualized support plan based on FBA	- Externalizing behavior - Implementation quality	- Indication of a significant positive effect of the N-PALS model for students with a persistently high-risk trajectory - N-PALS can moderate the development of externalizing behavior problems
Sørlie & Ogden (2007) [104]	N-PALS	S, T, P	Norway	QED (3 y)	T1 = Enhancement protective factors T2 = Behavioral + academic support, mentoring, social skills training T3 = Intensive and specific components for child and people around it	- Problem behavior in school - Problem behavior in school environment - Problem behavior in classroom - Behavior problematic students in class	- Moderate to large reductions in teacher observed problem behavior - Results of social competence and classroom climate were less encouraging - Implementation quality & teacher collective efficacy related to better outcomes
Sørlie & Ogden (2015) [80]	N-PALS	S, T, P	Norway	QED (3 y)	T1 = Prosocial skill training, systematic praise, classroom management strategies T2 = CICO T3 = Individualized support plan based on FBA	- Learning climate - Staff characteristics - Student body characteristics	- Positive effects on student problem behavior and classroom learning climate - Number of segregated students decreased in the intervention group, while it increased in the control group - Implementation quality moderated the outcomes
Sørlie, Ogden, & Røyrhus Olseth (2016) [81]	N-PALS	S, T, P	Norway	QED (3 y)	T1 = Prosocial skill training, systematic praise, classroom management strategies T2 = CICO T3 = Individualized support plan based on FBA	- Self-efficacy - Collective efficacy - Behavior management - Implementation quality	- Positive effect for collective efficacy, self-efficacy, and positive behavior support practices - Effects on student perceptions of teachers‚ behavior management strategies were, however, not consistent with the positive staff ratings.
Splett et al. (2017) [105]	ISF	S, T, P	USA	QED	T1 = School-wide mental health promotion	- social-emotional mental health interventions	- Proof of the main implementation components - Study shows characteristics of well-functioning ISF teams
Tobin & Sugai (2005) [106]	SWPBS	S, T, ST	USA	QED (2 y)	T1 = School-wide mental health promotion T2 = Social-emotional mental health interventions T3 = Social-emotional mental health interventions	- Effects on secondary and tertiary level - -Identifying problem behaviors - Self-control social skills	- Significant differences between the groups based on type of intervention received - Shows that SWPBS is an effective primary prevention intervention
Utley (2012) [107]	SWPBS	S, T, P	USA	QED	T1 = e.g., continuum of consequences for problem behavior T2 = e.g., system for increasing structure and predictability, contingent adult feedback T2 = e.g., reward system for appropriate behavior, continuum of consequences for problem behavior	- Implementation quality - Teacher level of cultural responsiveness (CR) in the classroom - Status of support	- Implementation fidelity was 90% - Examination of the CR - 50% of the teachers were culturally responsive. - 73% of them used culturally responsive teaching practices. - Only 43% used a variety of assessment methods to measure student achievement levels. - 90% of the teachers perceived the school to be a safer school environment.
Vainikainen, Hienonen, & Hotulainen (2017) [108]	Finnish three-tiered support model	S, T	Finland	RCT	T2 = Intensified support T3 = Special support	- Class size - Need of support	- Average larger classes perform better - Students receiving support study in slightly smaller classes - At the individual level, receiving support was related to lower initial performance. - At the class level, the proportion of students receiving support in the class predicted later performance positively
Vincent, Swain-Bradway, Tobin, & May (2011) [109]	SWPBS	S, T	USA	QED (3 y)	T2 & 3 (targeted students) = Individualized Education Plan	- ODR - Implementation quality	Discrepancy in discipline was present in schools that implemented SWPBS and schools that did not. - African-American students were over-represented among students with ODR. - In schools with SWPBS, the discipline gap was statistically significantly smaller.
Walker, Cheney, Stage, & Blum (2005) [110]	SPBS	S, T	USA	QED (3 y)	T1 = e.g., positive school-wide expectations taught and reinforced regularly T2 = existing school supports T3 = individualized behavior support	- ODR - Behavior - Social skills - Academic competence	- Students at risk for school failure are best identified by monitoring ODR and the use of a systematic school-wide screening process
Weiland, Murakami, Aguilera, & Richards (2014) [111]	PBIS	S, T	USA	MM (5 y)	n.a.	- ODR	- PBIS empowers teachers to maintain and manage many classroom behaviors that once would have resulted in an ODR. - Campus culture changed to a more positive environment. - Positive repercussions for students concerning academic success at higher grade levels
Wu et al. (2019) [77]	CW-FIT	S, T	Taiwan	MM (9w)	T1 = Reviewed three behavior expectations T 2 = Additional self-management	- Disruptive behavior	- CW-FIT is an effective intervention in increasing students on-task behaviors and decreasing disruptive behaviors. - Implementing multiple tiers of CW-FIT was much more effective than implementing solely Tier 1.
Yeung, Mary, Barker, & Brenda (2009) [112]	PBL	S, T	Australia	QED (9 m)	n.a.	- Cognitive self-concept - Affective school self-concept - English self-concept - Math self-concept - Parent self-concept - Effort goal orientation - Planning - Study management - Persistence	- Positive effects on school affective self-concept affective, English self-concept, parent self-concept, planning

n.a. = not available S = Students, P = Parents, T = Teacher, PST = Preservice teacher, ST = Staff, PS = Psychologist, SSW = Social school worker QED = Quasi experimental design, RCT = Randomized control trial, MM = Mixed method design, CS = Case study ODR = Office discipline referrals y = year, sy = school year, m = month, d = day T1, T2, T3, T4 = Tier 1, Tier 2, Tier 3, Tier 4.

**Table 2:** Tier 1 – tier 3 interventions.

Tiers	Interventions^a^
Tier 1	Schoolwide Rules or Trainings^1.2^, Direct Student Interventions^1.2^, Teacher Interventions^1.3^, Interventions related to Referrals^1.4^
Tier 2	Trainings & Supports^2.1^, Small Group Interventions^2.2^, Teacher Interventions^2.3^, Direct Student Interventions^2.4^, Consultancy^2.5^, Interventions related to Staff and Parents^2.6^, Individualized Support for Students^2.7^
Tier 3	Conflict related Interventions^3.1^, Intervention with Families^3.2^, Individualized Interventions^3.3^, Feedback^3.4^, Direct student interventions^3.5^, Training & Supports^3.6^, Teacher Interventions^3.7^

1.1 = Schoolwide Social Skill Training, Establishing Unified Attitudes, Schoolwide Mental Health Promotion, Broad Expectations.

Defining & teaching consistent behavioral expectations & recognizing students for expected and appropriate behaviors, School Rules, Classroom & building level approaches, Schoolwide Support for Student with/at Risk of EBD, Behavior Expectations.

1.2 = Token Economy System, Cool Tools, Gotcha Tickets, Strive for Five, Points/Praise/Goals, Direct Training Intervention Manual, Mentored Peer Mediation, Receiving Classroom Lessons, Academic Behavior Monitoring, Lessons by Homeroom teachers 1.3 = Teacher Training, Classroom Management Strategies, Explicit Instruction, Positive Reinforcement, Enhancement of Protective Factors, Sheltered Instruction Observation, Protocol (SIOP) Strategies, Feasible Strategies, Consolation, standards for quality teaching.

1.4 = Continuum of Consequences for problem behavior, Targeted Behavior Referrals.

2.1 = (Targeted) Social Skill Training, Anger Control Training, Behavioral and academic support, Using existing School supports.

2.2 = Time-limited small group instruction, Social Skills Group, Small group Intervention, Support Group, Formal & Informal Friendship Groups.

2.3 = Implementation Support Strategy, more Intensive Treatment Strategy, Implementation Planning, System for Increasing Structure and Predictability.

2.4 = Give Me Five, Tokensystems, Imagine It!, Behavior charts/contracts, Mentored Peer Mediation, Classroom Placement, CICO, Fostering Unified expectations, Self-Management, Cognitive Behavioral Interventions, Social-Emotional Mental Health intervention, Academic Interventions, Role Play.

2.5 = Applied Behavioral Analysis, Counseling.

2.6 = Parent Involvement, Contingent Adult Feedback.

2.7 = Classroom & Individualized Support, Individualized Education Plan, Individual Support Plan.

3.1 = Conflict Intervention, Problem Solving Training, Continuum of Consequences for Problem Behavior.

3.2 = Incorporates wraparound interventions for youth & families in crisis, Family & Interagency Collaboration, Intensive + specific components for child & people around it.

3.3 = Individual interventions & support plans, Behavior Intervention Plans (BIP), Individualized Intervention.

Functional Behavior Assessment (FBA), Intervention Plan for School Breaks, Function Based Intervention, Individualized Education Plan, Support in Small Groups, Differentiated Individual Support.

3.4 = Performance Feedback, Reward System for appropriate Behavior.

3.5 = CICO, Smaller Imagine It! Sessions, Interventions in Reading, Social-Emotional Mental Health Interventions.

3.6 = Social Skill Training, Intensive Support on Ongoing Basis, Special Education Support.

3.7 = Implementing Unified correction procedures, 1:1 Counseling, Participant Modeling sessions.

a Nine studies reported multilevel work but did not list explicit interventions and one study reported a fourth tier, which only focused on teacher behavior (Algozzine, Algozzine, 2007).

## 3 Results

### 3.1 Characteristics of multi-tiered systems of support in elementary education (RQ1 a,b,c)

#### 3.1.1 Interventions (RQ1 b)

Some studies were very explicit about which interventions were used, but nine studies simply stated that they were guided by the basic concept of the respective MTSS. The following upper categories for interventions could be derived from the data for the different tiers (Table 2):

For Tier 1, interventions such as school-wide rules or trainings, direct student interventions, teacher interventions, and interventions related to referrals were described. On Tier 2, trainings and supports, small-group interventions, teacher interventions, direct student interventions, consultancy, interventions related to staff and parents, and individualized support for students come into use. On Tier 3, the interventions could be summarized as conflict-related interventions, intervention with families, individualized interventions, feedback, direct student interventions, training and supports, and teacher interventions.

#### 3.1.2 Involved persons (RQ1 c)

The people involved include parents, school social workers, psychologists, other school staff, teachers, and students. In their conceptualizations, 75% of the MTSS include the three main groups of teachers, students, and parents. Parents are mentioned in 28% of the studies. A holistic, multimodal approach is postulated in various approaches. However, people from school systems seem to be less often integrated into the approaches and studies than people from within the school. Table 3 lists all types of involved persons integrated in the different types of MTSS.

**Table 3:** Frequencies of Involved Persons (evaluated and included in the concept).

Involved Persons Location										
	AUS	CAN	FIN	GER	NZ	NOR	TW	USA	total	%*
S, T	1		1				1	16	19	47.5%
S, PST								1	1	2.5%
S, T, P		1		1		4		3	9	22.5%
S, T, ST						1		2	3	7.5%
S, T, PS								2	2	5%
S, T, SSW								1	1	2.5%
S, T, P, ST					1			4	5	12.5%

S = Students, P = Parents, T = Teacher, PST = Preservice teacher, ST = Staff, PS = Psychologist, SSW = Social school worker *N = 40 = 100%.

Characteristics of Empirical Quantitative Research on Multi-tiered Systems of Support in Elementary Education (RQ2 a)

The publication period of the included studies ranged from 2004 to 2020. During this time, 40 studies have emerged internationally. Furthermore, 72.5% of all studies emerged between 2009 and 2016. During this period, 14 different MTSS approaches have been evaluated, but all of them relate to at least one of the three major overarching approaches SWPBS (5), PB(I)S (7), or RTI (4). Some MTSS approaches refer to multiple concepts as their origin. Accordingly, these subtypes cannot always be strictly delineated, and the transitions are fluid (Table 4).

**Table 4:** Overview MTSS from 2004 to 2020.

MTSS		Year																
		04	05	06	07	08	09	10	11	12	13	14	15	16	17	18	19	20
CW-FIT^1^	Class-wide function-related intervention teams															1	1	
EBI^1&2^	Evidenced-based Interventions			1														
FBA^2^	Functional Behavior Assessment													1				
Finnish MTSS^3^	Finnish three-tiered support model														1			
ISF^2^	Interconnected Systems Framework														1			
MTIS^2&3^	Multi-Tiered Implementation Supports												1					
N-PALS^1^	Positiv atferd, støttende læringsmiljø og samhandling				1								1	1		1		1
PBIS^2^	Positive Behavior Interventions and Support								1	2	1	3	1	1				
PBL^2^	Positive Behavior for Learning						1											
PBS^2^	Positive Behavior Support						1	1										
RIM^3^	Rügener Inklusionsmodell													1				
RTI^3^	Response to Intervention						1					1						
SWPBIS^1^	Schoolwide Positive Behavior Interventions and Support									2								
SPBS & SWPBS^1^	Schoolwide Positive Behavior Support	1	2	2	2	2			2	2								
total		1	2	2	3	2	3	1	3	6	1	4	3	4	2	2	1	1

^1^ = based on Schoolwide Positive Behavior Support.

^2^ = based on Positive Behavior Support.

^3^ = based on RTI.

Most studies (72.5%) came from the United States. Accordingly, this is where the largest research community is located. The other identified countries (AUS, CAN, FIN, GER, NOR, NZ, TWN) conducting research on the topic of MTSS provide one study, except for Norway, where five studies (12.5%) have emerged (Table 5).

**Table 5:** Overview MTSS & location.

MTSS		Location							
	AUS	CAN	FIN	GER	NOR	NZ	TWN	USA	total
CW-FIT^1^							1	1	2
EBI^1&2^								1	1
FBA^2^								1	1
Finnish MTSS^3^			1						1
ISF^2^								1	1
MTIS^2&3^								1	1
N-PALS^1^					5				5
PBIS^2^		1						8	9
PBL^2^	1								1
PBS^2^								2	2
RIM^3^				1					1
RTI^3^								2	2
SWPBIS^1^								2	2
SPBS & SWPBS^1^						1		10	11
total	1	1	1	1	5	1	1	29	40

^1^ = based on Schoolwide Positive Behavior Support (N = 21).

^2^ = based on Positive Behavior Support (N = 16).

^3^ = based on RTI (N = 6).

Twenty-six studies (65%) can be classified as a quasi-experimental design (QED), and they did not describe randomization in their sample. Six studies (15%) proceeded in the form of a (single)-case study with behavioral observations. In addition, three studies (7.5%) described a randomized control trial (RCT), and five studies (12.5%) used a mixed method approach consisting of quantitative and qualitative methods.

As seen in Table 1, many of the included studies are characterized by a very large sample, which also influences the quality of the study results: The studies often obtain their data from the school population registers and use only superficial evaluation methods. Often no detailed descriptive data and no interference statistics were described. Instead, percentage changes or differences between multiple groups are reported. Nevertheless, these studies extensively report positive outcomes of MTSS on student problem behavior. This is based on the number of Office Discipline Referrals (ODRs) transmitted by schools. Changing class structures or other influencing factors are often not included in the analyses. In addition, many of these studies do not report what interventions the different tiers held. However, they all report that MTSS were introduced and implemented.

#### 3.1.3 Outcome measures (RQ2 b)

Sixty percent of the studies focused on emotional and social behavior, 45% defined ODRs (or just referrals) as an outcome measure, and 32.5% surveyed implementation quality. Furthermore, 27.5% of the studies surveyed academic performance, and 12.5% surveyed school and classroom context (school safety, noise exposure, etc.), teachers', parents', and other stakeholders' perceptions of MTSS, and various explicit demographic information (e.g., socioeconomic status or mobility). In 10% of the studies, a general effect of the MTSS was inferred, and in 5%, the effectiveness with which certain groups were able to do their work in the MTSS was surveyed. More information and the underlying definitions of the coded variables can be found in Table 6.

**Table 6:** Outcome measures.

Outcome Measure	Definition	Studies	%*
Academic Performance	Variables that are directly related to academic performance.	11	27,5%
Demographic information	All variables that collect explicit demographic data as mobility or socioeconomic status.	5	12,5%
Efficacy	The variable surveyed was the efficiency with which the various groups involved in the MTSS performed their work.	2	5%
Emotional & Social Behavior	Constructs of emotional and social behavior were collected as variables.	24	60%
Implementation & Training	All Variables that collect information about the implementation quality and related trainings.	13	32,5%
Perception of Teachers, Parents & Staff	Variables that are directly related to perception of the persons involved in the MTSS.	5	12,5%
Referrals	Variables that are directly related to referrals or suspending.	18	45%
School/Classroom Context	Various constructs and mechanisms from the classroom or school context, such as noise exposure or school safety, were collected as variables.	5	12,5%
Total MTSS Effect	As variable, the effect of the entire MTSS was measured.	4	10%

Note that one study can have several outcome measures (e.g., Implementation & Staff Perception) *N = 40 studies (e.g., 18 of 40 studies included Referrals as Outcome Measure).

#### 3.1.4 Effects (RQ2 c)

More detailed results on behavior change are available for the (single-) case studies (Table 7). Six studies reported effect sizes and thus attributed different levels of effectiveness to MTSS. The studies conducted in Norway reported lower effectiveness than other studies. Nevertheless, the introduction of MTSS is discussed positively there. The six (single-) case studies report positive effects, especially about problem behavior. On-task behavior is often surveyed in connection with this.

**Table 7:** Single case studies behavior effects.

Study	Outcome Measure	Cases	Change (Mean)^a^	Significance
Franzen & Kamps (2008)[72]	Frequency of total student behaviors across 5-min sessions Frequency of active teacher supervision across 5-min sessions	180 10	G2: 15.54–5.95 G3: 12.92–6.91 G4: 12.06–6.73 G2: 2.87–5.20 G3: 2.10–6.10 G4: 1.89–6.13	n.a. n.a.
MacLeod et al. (2016) [73]	Problem behavior	4	S1: 41%–4% S2: 49%–2% S3: 45& – 14% S4: 26%–12%	n.a
Nelson et al. (2018) [74]	On-task behavior Praise rate (times) Reprimand rate (times)	20 22 24	C1: 59%–84% C2: 69%–90% C3: 69%–86% C1, 2, 3: 11.35–11.88 C1, 2, 3: 12.46–4.48	C1: *p* < .01 C2: *p* < .01 C3: *p* < .01 C1: *p* = .64 C2: *p* = .05 C3: *p* = .36 C1: *p* < .01 C2: *p* < .01 C3: *p* = −1.04
Pearce (2009) [75]	Number of maladaptive episodes	9	Positive effects from the graphs are discussed, but no statistical data are given.	n.a.
Sanetti & Collier-Meek (2015) [76]	Number of disruptive behavior	6	S1: 10.08–8.01 S2: 12.00–12.32 S3: 12.35–11.97 S4: 11.47–8.15 S5: 6.65–4.66 S6: 2.00–0.73	n.a
Wu et al. (2019) [77]	On-task behavior Disruptive behavior	1	21%–72% 78%–35%	n.a n.a.

a = Percent rounded due to representation S = Student G = Grade C = Classroom.

Another six studies (Table 8) reported effects in terms of Cohen's d (one study reported interaction effects). Again, problem behavior is the focus of the surveys, and it reached effect sizes between 0.11 and 0.94 (disrupting class). In addition, the studies more frequently surveyed class climate (0.01–0.69) and on and off task behavior. One study also surveyed prosocial behavior (0.36–0.51) and social integration (0.36). All other studies reported no effects, but only changes in the form of group differences or percentage changes between different groups.

**Table 8:** Studies effects behavior.

Study	Outcome Measure	Effect (Cohen's d)	Significance
Algozzine et al. (2007) [78]	Answering questions	1.06	n.a
	Talking about academics	0.59	
	Paying attention	1.22	
	Raising hand	1.43	
	Total on-task/appropriate	1.63	
	Disrupting class	0.94	
	Looking around	1.14	
	Talking inappropriately	1.37	
	Doing inappropriate task	1.20	
	Total off-task	1.63	
Borgen et al. (2020) [79]	Classroom noise	−0.057	n.a
	Bullied	0.0058	
	Well-being	−0.026	
	Special education	0.0027	
Sørlie & Ogden (2015) [80]	Problem behavior on common school areas	0.25	p = .001
	Moderate problem behavior	0.24	p = .001
	Serious problem behavior	0.17	p = .033
	Problem behavior in classroom	0.13	p = .092
	Moderate problem behavior	0.12	p = .110
	Serious problem behavior	0.11	p = .162
	Classroom climate (staff)	0.17	p = .017
	Classroom climate (students)	0.01	p = .761
	Student relations	0.10	p = .633
	Teacher relations	0.00	p = .984
Sørlie et al. (2016) [81]	Collective efficacy	0.34	p = .000
	Self-efficacy	0.14	p = .014
	Positive behavior support (staff)	0.91	p = .000
	Positive behavior support (students)	0.01	p = .709
	Behavioral correction (staff)	0.01	p = .943
	Behavioral correction (students)	0.05	p = .167
Blumenthal & Voβ (2016) [82]	Problem behavior	−0.39^b^	p = .000
		−0.43^c^	p = .000
	Prosocial behavior	0.36^b^	p = .000
		0.50^c^	p = .000
		0.51^d^	p = .005
	Feeling of being accepted	0.35^c^	p = .004
	Classroom climate	0.29^c^ 0.69^d^	P = .011 P = .002
	Self-concept of school readiness Social integration	0.38^c^ 0.36^c^	P = .000 p = .002
Cheney, Blum, & Walker (2004) [83]	Social skills Problem behaviors	F = 3.0/5.1^a^ F = 0.1/5.7^a^	p < .05/p < .009 p < .99/p < .005

a Main effect/Interaction effect.

b = Children without underperformance.

c = Children with low underperformance.

d = Children with severe underperformance.

## 4 Discussion

The aim of the review, to provide an overview of different areas of MTSS in elementary schools with a focus on behavior modification, was largely achieved. Statements can be derived about the scope of the studies, the studies' location, conceptual relationships between different forms of MTSS, the study design, and the research methodology, including effects. The first part of the research questions (RQ1) attempts to compare and classify the different characteristics of MTSS internationally, whereas the results of the second research questions (RQ2) provides a current state of international empirical quantitative research on MTSS.

First, a wide range of different MTSS forms (15) with different locations (8) can be discussed as results of the review. Some studies are also part of other reviews, but the studies presented here can be seen as an extension of the existing reviews [5,[10], [11], [12], [13], [14], [15]]. Internationally, the various MTSS forms and their origins or references are clearly attributable to the U.S. research and education community. Considering the results, it can be concluded that MTSS systems that include behavior modification interventions in elementary school are more likely to have a Western reference and origin. This is also the result of a search matrix with terms of a Western-influenced school research.

Furthermore, the various interventions described and used all focus primarily on the students. This is due to the origin of the approaches. A conceivable extension would be additional interventions on the teacher level. An exemplary approach could be accompanied by coaching or counseling sessions (supervision) for the teachers, which are oriented toward the needs and circumstances of the students and the teachers. This would also strengthen the systemic nature of MTSS further.

In this context, the conviction, and the transport of the system on the part of the school management and the community of teachers should also be mentioned. Studies have shown that more convinced teachers and more committed school administrators were able to implement MTSS better and more sustainably [94,113]. This point underlines the flexibility that the system must provide to be successful, which should be considered in the development and description of the MTSS approach.

Concerning the involved groups of persons, it is striking that about 75% of the studies researched teachers, students, and parents, and only the remaining 25% also included other school staff, psychologists, and school social workers in the sample. The holistic approach of MTSS often does not do justice to the study designs in this regard. Another point in this context is that only 28% of the studies included parents in the research. Especially, it is known that parental influence on education can be significant when combined with school-based interventions [e.g., [114,115]]. An explanation for this may be the often costly and extensive scientific monitoring of the multimodal approach of MTSS. In addition, there are a higher number of evidence-based surveys that ask about school experiences at the teacher or student level than for the parent perspective or that of other staff.

Then the time frame of the studies included in this review should be mentioned. The first study that meets the selection criteria is from 2004 [83], which may be explained by the policy specifications in the United States. For example the term *Positive Behavioral Interventions and* Supports is used prominently in the Individuals with Disabilities Education Improvement Act of 2004 [116]. This also underscores the increasing political importance of MTSS at this time and may explain the increase in studies after 2004. This is also directly related to the dissemination of the studies in international comparison. It is important to note that the search was only conducted in German and English. Nevertheless, it can be concluded that the prevalence of MTSS in an international comparison is strongly focused on the USA. However, this is again due to legal regulations and educational policy work. The statement about the international comparison of different forms is therefore slightly diminished. Another important factor is the fact that only studies were included that focused exclusively on behavior or the combination of behavior-related interventions and learning-related interventions. All results must be considered against this background. However, this is by no means a limitation, but it should be kept in mind when looking at the results.

The second part of the research questions (RQ2) focused mainly on characteristics of empirical quantitative research on MTSS in elementary education internationally. In this context it is critical to note that while there are many studies on MTSS, they rarely address the effectiveness of MTSS as an overall system and the effectiveness of the individual stages in elementary school. While results from individual schools or classrooms are consistently reported, most studies are limited to ODRs as an indicator of problem behavior. Moreover, one study criticized this approach [100]. The authors concluded that using ODR as a baseline for intervention planning is not very meaningful. Other studies reach different conclusions and emphasize validity in ODRs as a reference point for intervention planning [e.g., [117]]. An interesting and informative method in this context are studies in single-case design [[118], [119], [120]]. Various interventions, such as Check-In/Check-Out or the Good Behavior Game, have been tested for effectiveness in this manner [e.g., [[121], [122], [123]]]. A single-case design, as practiced in some of the studies included here, would be useful for answering some more detailed research questions. Single-case studies have become a proven research method to identify effects independent of a norm sample [124]. The analysis of the data presented of studies included in this review was mostly descriptive. Here, a more precise and meaningful evaluation in terms of effects (e.g., hierarchical piecewise regression) would be appropriate. Moreover, interpretable effects can be traced along the change in behavior of specific individuals [125]. The case studies in this review identify the effect only in terms of change. For this, the authors used the mean of the baseline measure, which was compared to the intervention phase afterwards. In addition, it is often not fully explained how exactly the research is designed and how often and when teachers observed the behavior. Due to the high informative value of this type of study, especially about behavior change, slight research desiderate arises here.

Looking at the various outcome measures on which the studies were based, it can be summarized that a majority of these focused on Emotional and Social Behavior and Referrals. Here again, the search matrix becomes relevant. It is also critical to note that the effectiveness of individual groups or the entire MTSS is only considered in about 15% of the studies. Thus, effectiveness is often measured directly by student outcomes. Due to the complexity of such systems and the many influencing factors, a multidimensional examination of several perspectives seems to make sense.

Subsumed MTSS can are described as effective, both on the teacher level but also on the side of the students. This becomes particularly clear when the results of the various studies are compared. Thus, six studies report various effects of MTSS on different subdomains, such as problem behavior, prosocial behavior, classroom climate, social integration, on-task and off-task behavior, and self-efficacy. The greatest effects can be found around behavioral changes (d = 0.11–0.94). One study [82] distinguished between children without underperformance, children with low underperformance, and children with severe underperformance. Here, the effects were greater for children with strong underperformance than for children with slight or no underperformance. The study results of the Norwegian and Finnish research groups are diverse. The entire MTSS is assigned a high effectiveness, but no significant effects (except for moderate problem behavior and collective efficacy) could be found [[79], [80], [81]].

### 4.1 Limitations

First, the validity of the systematic literature review is limited by the restriction of the sample to the elementary school sector. The statements cannot be transferred to other school types unrestrictedly. In addition, the included studies involve many different age groups (kindergarten through seventh grade) despite being limited to the elementary school sector. Another issue is the selection of studies based on the criterion of “academic learning.” All studies that focused only on academic learning and did not include behavioral dimensions were excluded. Since MTSS was originally strongly characterized by interventions in academic learning, large studies that play a relevant role in relation to the debate regarding the introduction of MTSS in educational systems could be dropped out here. In addition, studies whose sample was not explicitly described were excluded. In this case, it was assumed that it would not be a pure elementary school sample, but this did not affect many studies. In this context, the difficulty of defining elementary school is another limitation of the review, as there is no international, consistent definition. Therefore, a little tolerance must be shown toward the samples included. They are described as precisely as possible for this reason. Other limitations include search strategies and study selection. Only studies that passed a peer review process and were also published in English or German language were included. Since research on MTSS is often politically connoted and socially motivated, it is obvious that such studies are published in the respective national language, even more if they serve the further development and research of the local educational system. Certainly, there are other studies that have emerged internationally that would meet the criteria of this review but were not included due to language barriers. Another methodological criticism is that a metanalytic approach would be conceivable and certainly helpful for comparing effect sizes. However, the approach chosen here to report effect sizes and compare them descriptively is certainly not mistaken and less informative, especially since the variables collected often vary considerably. In addition, the samples, and the settings as well as basic concepts of the different MTSS forms vary greatly.

## 5 Conclusion

The effectiveness of MTSS, especially in elementary education, could be confirmed by this review. However, this more detailed insight into the quality of the studies also revealed some gaps which are presented below: The first gap that could be filled by research designs examining different tiers and their mechanisms of action, as well as the interaction of different interventions. In this way, a higher significance of the studies could be guaranteed. In this case, the use of daily behavioral ratings over a longer period could be conceivable since these are characterized by a high density of information regarding the different levels of support and the associated effects on behavioral changes. The use of multilevel analyses should also be emphasized here due to the mostly nested samples. It is complicated to investigate MTSS statistically and to precisely describe the connection of the different levels and the effects of the different teachers.

Another point is the extensions of interventions on the teacher side (as well as school social workers, psychologists, or other staff) which also could further strengthen the MTSS coherence. In this context, the development process of the MTSS must be considered as well: It would be conceivable to involve all groups of people already in the development of the approaches. Various studies have shown a significantly higher MTSS adaptation in relation to the problems to be addressed, and it can also prove that the success with which a system is implemented depends on the attitude that accompanies it [113]. In practical terms, this means that the development of school programs should be a joint effort between educational institutions, ongoing diagnostic training, and a flexible approach to the ever-evolving school system. This includes active collaboration with external organizations and internal staff, as numerous studies have shown [83,126]. In inclusive schools in particular, the cooperation between educational experts and psychologists or counselors is crucial for the successful implementation of MTSS [78].

In the context of professionalization, the implementation of measures and the establishment of an MTSS in a school system are also challenges that some of the studies address. These primarily have an educational policy dimension. The successful implementation of measures and systemic approaches depends largely on resources and training processes. Training processes that further enhance implementation quality should be considered in terms of the feasibility and fidelity with which an MTSS is implemented. Here, for instance, multiplier concepts and close support and advice from school authorities are indispensable. It is therefore clear that MTSS is more than a multi-tiered support concept, also having a political dimension that affects all areas of implementation and sustainability.

## Author contribution statement

All authors listed have significantly contributed to the development and the writing of this article.

## Data availability statement

Data will be made available on request.

## Declaration of competing interest

The authors declare that they have no known competing financial interests or personal relationships that could have appeared to influence the work reported in this paper.
