# Supplementary material for: Multi-tiered systems of support with focus on behavioral modification in elementary schools: A systematic review
Source: Heliyon. 2023 Jun 22;9(6):e17506. doi: 10.1016/j.heliyon.2023.e17506 (PMC10319208; doi:10.1016/j.heliyon.2023.e17506)
Supplement: Multimedia component 1 [file mmc1.docx]

**Supplement for: Multi-tiered Systems of Support with Focus on Behavioral Modification in Elementary Schools: A Systematic Review**

The variables listed here with their respective definitions were used in the underlying article. These variables were coded by the project team during the extraction process of the systematic literature review.

| **Variable** | **Definition** |
| --- | --- |
| Academic Performance | Variables that are directly related to academic performance |
| Case | Case of the Single Case Study |
| Change (Mean) | Described Change of the different cases |
| Demographic information | All variables that collect explicit demographic data as mobility or socioeconomic status |
| Design | Study design along: Single Case Study, Randomized Control Trial, Quasi Experimental Design, Mixed Method |
| Effect | Described Effect of the different cases |
| Efficacy | The variable surveyed was the efficiency with which the various groups involved in the MTSS performed their work |
| Emotional & Social Behavior | Constructs of emotional and social behavior were collected as variables |
| Implementation & Training | All Variables that collect information about the implementation quality and related trainings |
| Interventions | Interventions of the MTSS Form described in the study |
| Involved Persons | Persons involved in the MTSS (Students, Teachers, Parents, Staff, School Social Workers, Psychologists, Preservice Teachers) |
| Location | Location of the study |
| Main Results | Main Results of the Study described in the Abstract or results/discussion of the study |
| MTSS Form | Name and Abbreviation of the MTSS |
| Outcome Measures | Outcome Measures of the MTSS Form described in the study |
| Perception of Teachers, Parents & Staff | Variables that are directly related to perception of the persons involved in the MTSS |
| Reference | Authors Reference |
| Referrals | Variables that are directly related to referrals or suspending |
| School/ Classroom Context | Various constructs and mechanisms from the classroom or school context, such as noise exposure or school safety, were collected as variables |
| Significance | Describes Significance of the different cases |
| Total MTSS Effect | As variable, the effect of the entire MTSS was measured |

MTSS = Multi-tiered Systems of Support
